# Cytoskeletal remodeling slows cross‐bridge cycling and ATP hydrolysis rates in airway smooth muscle

**DOI:** 10.14814/phy2.14561

**Published:** 2020-08-18

**Authors:** Philippe Delmotte, Young‐soo Han, Gary C. Sieck

**Affiliations:** ^1^ Department of Physiology and Biomedical Engineering Mayo Clinic Rochester MN USA

**Keywords:** actin polymerization, cytoskeletal remodeling, tension cost

## Abstract

During isometric activation of airway smooth muscle (ASM), cross‐bridge cycling and ATP hydrolysis rates decline across time even though isometric force is sustained. Thus, tension cost (i.e., ATP hydrolysis rate per unit of force during activation) decreases with time. The “latch‐state” hypothesis attributes the dynamic change in cross‐bridge cycling and ATP hydrolysis rates to changes in phosphorylation of the regulatory myosin light chain (rMLC_20_). However, we previously showed that in ASM, the extent of rMLC_20_ phosphorylation remains unchanged during sustained isometric force. As an alternative, we hypothesized that cytoskeletal remodeling within ASM cells results in increased internal loading of contractile proteins that slows cross‐bridge cycling and ATP hydrolysis rates. To test this hypothesis, we simultaneously measured isometric force and ATP hydrolysis rate in permeabilized porcine ASM strips activated by Ca^2+^ (pCa 4.0). The extent of rMLC_20_ phosphorylation remained unchanged during isometric activation, even though ATP hydrolysis rate (tension cost) declined with time. The effect of cytoskeletal remodeling was assessed by inhibiting actin polymerization using Cytochalasin D (Cyto‐D). In Cyto‐D treated ASM, isometric force was reduced while ATP hydrolysis rate increased compared to untreated ASM strips. These results indicate that external transmission of force, cross‐bridge cycling and ATP hydrolysis rates are affected by internal loading of contractile proteins.

## Graphical abstract

An increase in internal loading of the contractile proteins resulting from actin cytoskeletal remodeling during isometric activation is associated with a decrease in cross‐bridge cycling rate and ATP hydrolysis rate.

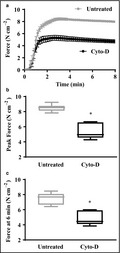

## 1 INTRODUCTION

Airway smooth muscle (ASM) force generation results from cross‐bridge recruitment and cycling driven by ATP hydrolysis (Gunst & Fredberg, 2003; Jones, Lorenz, Prakash, Sieck, & Warner, 1999a; Seow, 2005; Sieck, Han, Prakash, & Jones, 1998), which underlies the fundamental contractile mechanism for all forms of muscle. During isometric activation of ASM, cross‐bridge cycling and ATP hydrolysis rates rapidly increase reaching a peak before decreasing even though isometric force is sustained (Dogan, Han, Delmotte, & Sieck, 2017; Jones, Lorenz, et al., 1999; Jones, Perkins, et al., 1999). Thus, tension cost (i.e., ATP hydrolysis rate per unit of force during activation) declines over time. The “latch” state hypothesis attributes the dynamic change in cross‐bridge cycling and ATP hydrolysis rates to dephosphorylation of the regulatory myosin light chain (rMLC_20_) (Fredberg et al., 1996; Hai & Murphy, 1988a, 1988b; Murphy & Rembold, 2005). However, we previously showed that the extent of rMLC_20_ phosphorylation remains unchanged during sustained isometric force in canine ASM (Jones, Lorenz, et al., 1999).

It is well known that both cross‐bridge cycling and ATP hydrolysis rates are affected by loading of contractile proteins (Jones, Lorenz, et al., 1999). Internal loading of contractile proteins is imposed by cytoskeletal remodeling (involving actin polymerization) and the tethering of contractile elements through dense bodies to the cortical cytoskeleton and ASM membrane to allow force transmission (Chitano et al., 2017; Dogan et al., 2017; Gunst & Fredberg, 2003; Gunst & Tang, 2000; Gunst, Tang, & Opazo, 2003; Gunst & Zhang, 2008; Jones, Perkins, et al., 1999; Mehta & Gunst, 1999; Seow & An, 2020; Tang, 2018; Wang, Wang, & Tang, 2020; Zhang & Gunst, 2008). Actin exists in a monomeric globular (G) form that polymerizes to filamentous (F) actin during ASM force generation (Hirshman & Emala, 1999; Jones, Perkins, et al., 1999; Mehta & Gunst, 1999). We and others previously showed that disruption of actin polymerization greatly reduces ASM force generation (Jones, Perkins, et al., 1999; Mehta & Gunst, 1999), which likely reflects a disruption of the tethering of contractile elements to the cortical cytoskeleton and ASM membrane. Previous studies have shown that cytoskeletal proteins involved in the tethering of actin filaments to the cortical cytoskeleton and ASM membrane such as vinculin or focal adhesion protein paxillin contribute to ASM force during agonist stimulation but do not affect rMLC_20_ phosphorylation (Opazo Saez et al., 2004; Tang, Wu, Opazo Saez, & Gunst, 2002).

We hypothesized that an increase in internal loading of the contractile proteins results from actin cytoskeletal remodeling during isometric activation, which slows cross‐bridge cycling and ATP hydrolysis rates. To test this hypothesis, we examined the impact of cytochalasin D (Cyto‐D) inhibition of actin polymerization on simultaneously measured isometric force and ATP hydrolysis rate in permeabilized porcine ASM strips during maximum Ca^2+^ activation. Tension cost of ASM contractile activation was calculated as the ratio of ATP hydrolysis rate to isometric force and changes in rMLC_20_ phosphorylation were determined.

## 2 MATERIAL AND METHODS

### 2.1 Porcine ASM Strip preparation

Porcine tracheas were obtained from local abattoir and immediately immersed in chilled physiologic saline solution (PSS; composition in mM: 118.9 NaCl, 1.2 MgSO_4_, 1.2 KH_2_PO_4_, 4.7 KCl, 2.5 CaCl_2_, 0.03 EDTA, 5.5 dextrose, 25.0 HEPES). Porcine ASM strips (0.5 mm wide and 4–5 mm long) were isolated as previously described (Dogan et al., 2017; Jones, Lorenz, et al., 1999; Sieck, Dogan, Young‐Soo, Osorio Valencia, & Delmotte, 2019; Sieck, Han, Pabelick, & Prakash, 2001; Sieck et al., 1998; Sieck, Kannan, & Prakash, 1997). Porcine ASM strips were permeabilized with 10% (v/v) Triton X‐100 (Sigma‐Aldrich Co., Germany) in pCa 9.0 for 30 min for the simultaneous measurement of ATP hydrolysis rate and isometric force, actin polymerization, and rMLC_20_ phosphorylation assessments.

### 2.2 Simultaneous measurements of ATP hydrolysis rate and isometric force

**Figure 1:**
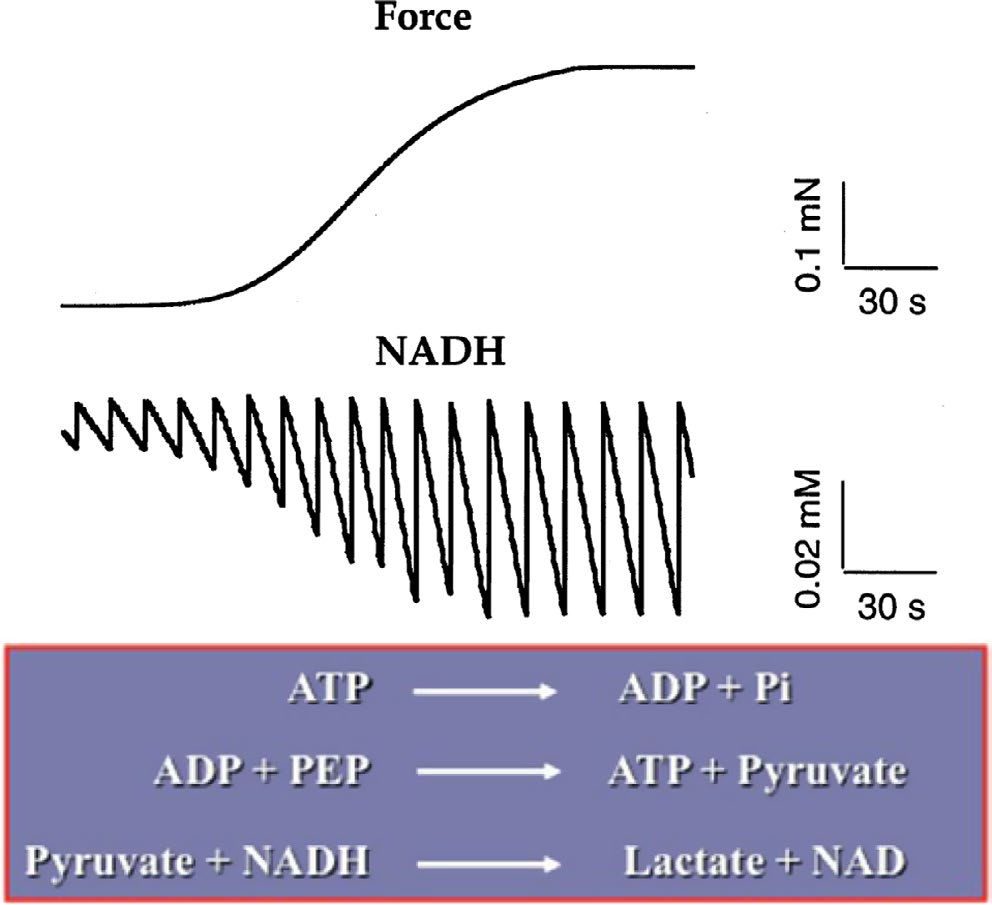
Simultaneous measurement of ASM force and ATP consumption rate (using NADH fluorescence) in permeabilized porcine ASM strips during maximum Ca^2+^ activation (pCa 4.0)

In permeabilized porcine ASM strips (10% Triton‐X 100 in pCa 9.0 for 30 min) treated and untreated with Cyto‐D (1 µM in pCa 9.0 for 10 min), ATP hydrolysis rate was measured using an enzymatic‐coupled β‐NADH fluorescence technique in a Guth Muscle Research System as previously described (Figure 1) ( Dogan et al., 2017; Jones, Lorenz, et al., 1999; Sieck & Gransee, 2012; Sieck et al., 1998). Fluorescence and force signals were simultaneously measured. The ASM strips were mounted in a flow through quartz cuvette on the system where one end of the strip was attached to a micrometer for length adjustment and the other end was attached to a force transducer. The quartz cuvette was continuously perfused with relaxing (pCa 9.0) or activating (pCa 4.0) solutions. A computer algorithm (described by Fabiato & Fabiato (1979) with stability constants listed by Godt & Lindley, (1982)) was used to determine the activating (pCa 4.0) and relaxing (pCa 9.0) solutions. The solutions contained the following (in mM): 7.0 EGTA, 1.0 free Mg^2+^, 5.0 MgATP, and 70.0 imidazole. The solutions also contained 1 μm calmodulin and pH was buffered to 7.1 with proprionic acid while ionic strength was kept constant at 150 mM by adjusting the concentration of potassium proprionate at 0.150 M. For the activating pCa 4.0 solution, 0.1 mM CaPr_2_ was also added. In addition to these constituents, solutions for ATP hydrolysis rate measurements also contained 5 mM phosphoenol pyruvate, 0.18 mM NADH, 140 U ml^−1^ lactate dehydrogenase, and 100 U ml^‐1^ pyruvate kinase. ATP hydrolysis rate was coupled to the oxidation of NADH (fluorescent compound) to NAD^+^ (nonfluorescent compound) (Figure 1). For each mole of ADP produced, 1 mol of NADH is oxidized to NAD^+^. The perfusion of the quartz cuvette was stopped for a 15‐s period during which decrease in NADH fluorescence was measured as a reflection of ATP hydrolysis rate in the ASM strips. The basal level of ATP hydrolysis was measured during exposure to a relaxing solution at pCa 9.0. Thereafter, the ASM strip was activated by exposure to a pCa 4.0 solution and force and ATP hydrolysis rate were measured and normalized for tissue volume. The ratio of ATP hydrolysis rate to force during activation at pCa 4.0 was used to calculate tension cost.

#### 2.2.1 Assessment of Actin Polymerization

Actin polymerization in porcine ASM was determined as described previously (Dogan et al., 2017; Jones, Perkins, et al., 1999; Sieck et al., 2019; Tang & Gunst, 2004) using a G‐/F‐actin in vivo assay kit (Cytoskeleton Inc., Denver, CO). Briefly, one pair of permeabilized ASM strips were treated with 1 µM Cyto‐D or left untreated in pCa 9.0 for 10 min at 22°C and another pair of permeabilized ASM strips were treated with 1 µM Cyto‐D or left untreated in pCa 9.0 for 10 min then stimulated with pCa 4.0 solutions for 10 min at 22°C. The ASM strips (4 per trachea) were then snap‐frozen. Subsequently, the ASM strips were thawed at room temperature and then minced in F‐actin stabilization buffer (composition in mM; 50 PIPES pH 6.9, 50 KCl, 5 MgCl_2_, 5 EGTA, 5% (v/v) Glycerol, 0.1% Nonidet P40, 0.1% Triton X‐100, 0.1% Tween 20, and 0.1% 2‐mercapto‐ethanol, supplemented with 1mM ATP and 1% protease inhibitor cocktail). Separation of F‐ and G‐actin was performed using standard western blotting technique with incubations of primary rabbit polyclonal anti‐actin antibody (1:1,000 dilution) (Cytoskeleton Cat# AAN01, RRID:AB_10708070) and secondary peroxidase AffiniPure goat anti‐rabbit HRP IgG (1:10,000 dilution) (Jackson ImmunoResearch Labs Cat# 111–035–144, RRID:AB_2307391). The actin bands were analyzed with a ChemiDoc MP Imaging System (Bio‐Rad Laboratories, Hercules, California, U.S.).

#### 2.2.2 rMLC_20_ Phosphorylation

Six pairs of Cyto‐D treated (1 µM in pCa 9.0 for 10 min) or untreated permeabilized ASM strips from six animals (*n* = 6) were stimulated with pCa 4.0. After 0, 0.5, 1, 2, 4, 6, and 8 min of activation, phosphorylation of rMLC_20_ was assessed using standard western blot technique as described previously (Dogan et al., 2017).

Protein extraction was performed in RIPA lysis buffer (Cell Signaling Technology, Danvers, MA) supplemented with 1 mM phenyl methane sulphonyl fluoride (PMSF), 1x phosphatase inhibitor (PhosSTOP Easypack Roche, Germany) and 1x protease inhibitor (cOmplete Mini Roche, Germany). Protein samples from Cyto‐D treated and untreated groups were separated on the same 15% SDS gel (20 µg per well) and transferred to the polyvinylidene fluoride (PVDF) membranes. Membranes were probed with a specific antibody for p‐rMLC_20_ (phospho S20) (1:1,000 dilution) (Cat# Ab2480, RRID:AB_303094, Abcam, Cambridge, MA) (Dogan et al., 2017). Membranes were then stripped and probed again for total rMLC_20_ (1:1,000 dilution, Monoclonal Anti‐Myosin Light Chain, Clone MY‐21, Cat# M4401, RRID:AB_477192, Sigma‐Aldrich Co.) (Dogan et al., 2017). p‐rMLC_20_ bands and total rMLC_20_ and were visualized using an enhanced chemiluminescence (ECL) technique and quantified using a ChemiDoc MP Imaging System (Bio‐Rad Laboratories). GAPDH (Cat# G9545, RRID:AB_796208, Sigma‐Aldrich Co.) was used as loading control. Data are presented as the ratio of p‐rMLC_20_ to total rMLC_20_.

### 2.3 Statistical Analysis

Experiments were carried out in a paired matched (Cyto‐D treated or untreated) manner using ASM strips isolated from porcine tracheas of 5–6 animals. A two‐way ANOVA was used to compare time‐dependent changes in force, ATP hydrolysis rate, tension cost, and rMLC_20_ phosphorylation in Cyto‐D treated and untreated ASM strips. Data are presented as means ± *SD* or box and whiskers (10–90 percentiles). Significance was considered at *p* < .05.

## 3 RESULTS

**Figure 2:**
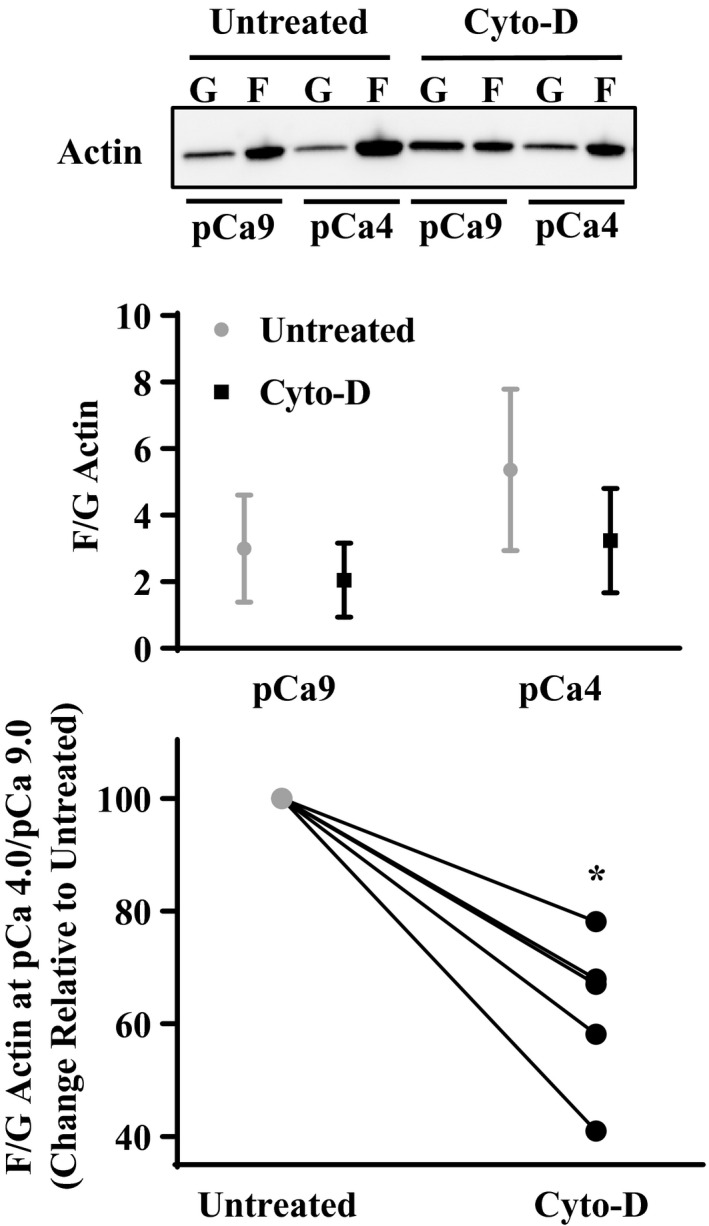
Effect of Cyto‐D on F‐ to G actin ratio in permeabilized porcine ASM strips. Representative western blots for F‐ and G‐ actin content in permeabilized porcine ASM strips that were untreated or treated for 10 min to 1 µM Cyto‐D in pCa 9.0 solution followed by maximal Ca^2+^ activation (pCa 4.0) for 10 min. In untreated ASM, the ratio F‐ to G‐ actin was significantly increased after pCa 4.0 activation. Actin polymerization after pCa 4.0 activation was greatly decrease by Cyto‐D treatment (decrease in F‐ to G‐actin ratio) compared to untreated ASM strips. *Significant difference (*p* < .05) compared to untreated ASM strip (*n* = 5)

In permeabilized porcine ASM strips treated for 10 min with 1 µM Cyto‐D in a pCa 9.0 solution, the ratio of F‐ to G‐actin at baseline (pCa 9.0 solution) was not significantly different compared to untreated ASM strips (*p* > .05, *n* = 5; Figure 2). During Ca^2+^ activation (pCa 4.0 solution), the F‐ to G‐actin ratio increased compared to baseline (pCa 9.0 solution) in both Cyto‐D treated and untreated ASM strips but the increase in F‐ to G‐actin ratio was greater in untreated compared to Cyto‐D treated ASM strips (*p* < .05, *n* = 5; Figure 2) showing that Cyto‐D treatment effectively reduces basal actin polymerization.

**Figure 3:**
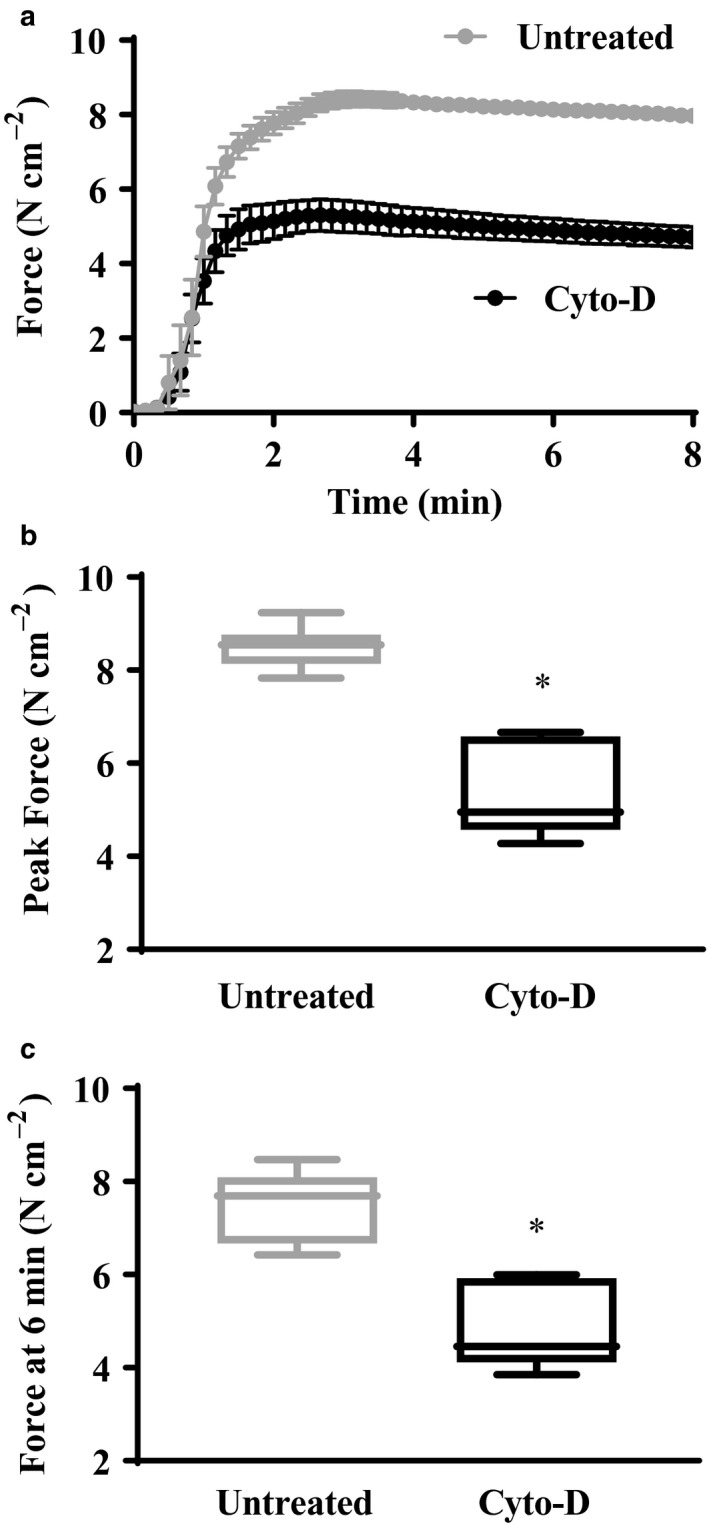
Effect of Cyto‐D on isometric force generation in permeabilized porcine ASM strips during maximal Ca^2+^ activation (pCa 4.0). The generation of isometric force in untreated control (open circles; *n* = 6) and Cyto‐D (1 µM) treated (filled circles; *n* = 6) ASM strips varied with time reaching a peak after 1–2 min and then decreasing to a steady state after ~ 5–8 min (a). Both peak force (b) and steady‐state force (c) were significantly reduced in Cyto‐D treated ASM strips *Significant difference (*p* < .05) compared to untreated ASM strip (*n* = 6)

In permeabilized porcine ASM strips, isometric force induced by maximal Ca^2+^ activation (pCa 4.0) initially reached peak values within 1–2 min then slowly declined throughout activation (Figure 3a). In ASM strips treated for 10 min to 1 µM Cyto‐D, the peak values for isometric force were greatly reduced compared to untreated ASM strips (*p* < .05, *n* = 6) (Figure 3b). Similarly, values for isometric force at 6 min were greatly reduced in ASM strips treated with Cyto‐D compared to untreated ASM strips (*p* < .05, *n* = 6) (Figure 3c).

**Figure 4:**
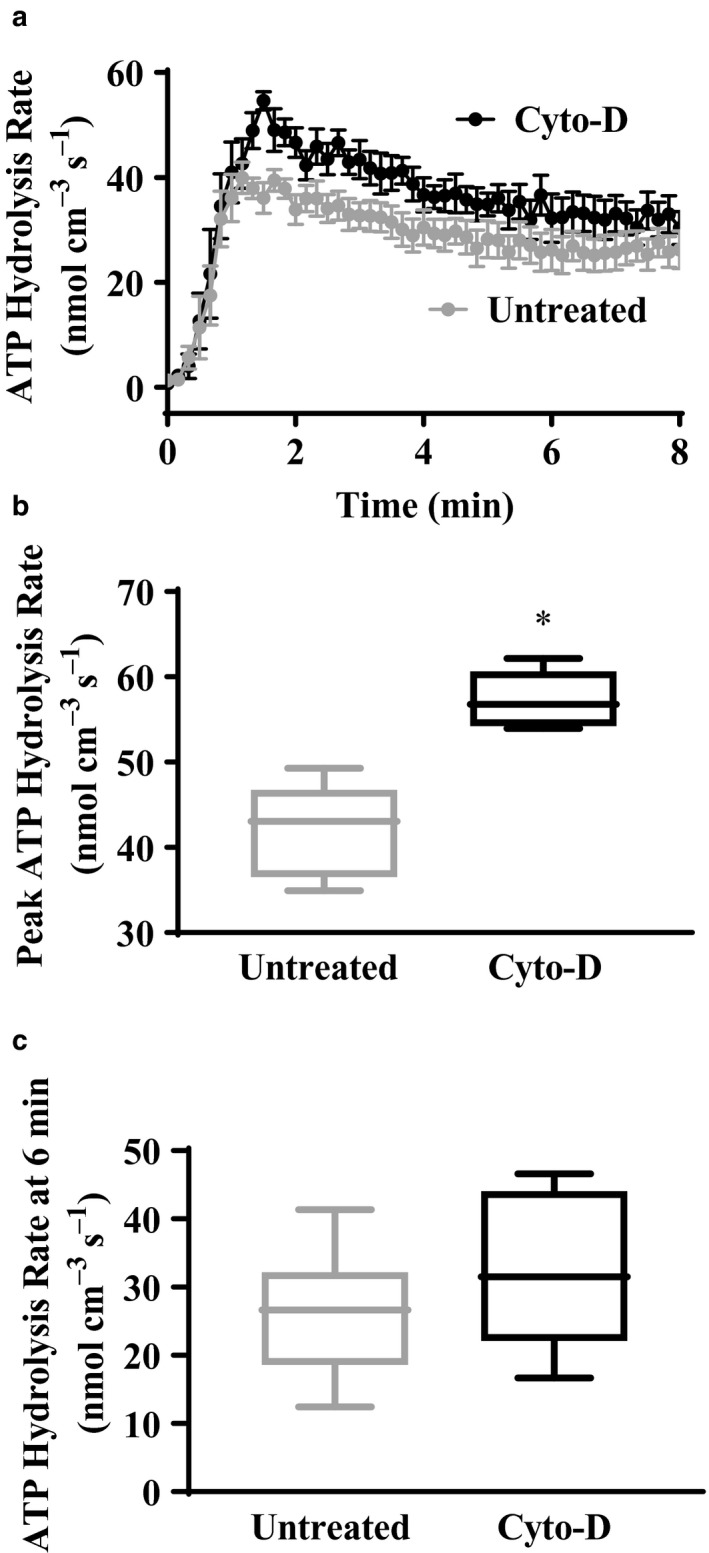
Effect of Cyto‐D on isometric ATP hydrolysis rate in permeabilized porcine ASM strips during maximal Ca^2+^ activation (pCa 4.0). Isometric ATP hydrolysis rate in untreated control (open circles; *n* = 6) and Cyto‐D (1 µM) treated (filled circles; *n* = 6) ASM strips varied with time reaching a peak after 1–2 min and then decreasing to a steady state after ~ 5–8 min (a). Peak ATP hydrolysis rate was significantly reduced in Cyto‐D treated ASM strips (b; *p* < .05), whereas steady‐state ATP hydrolysis rate was similar between groups (c). *Significant difference (*p* < .05) compared to untreated ASM strip (*n* = 6)

**Figure 5:**
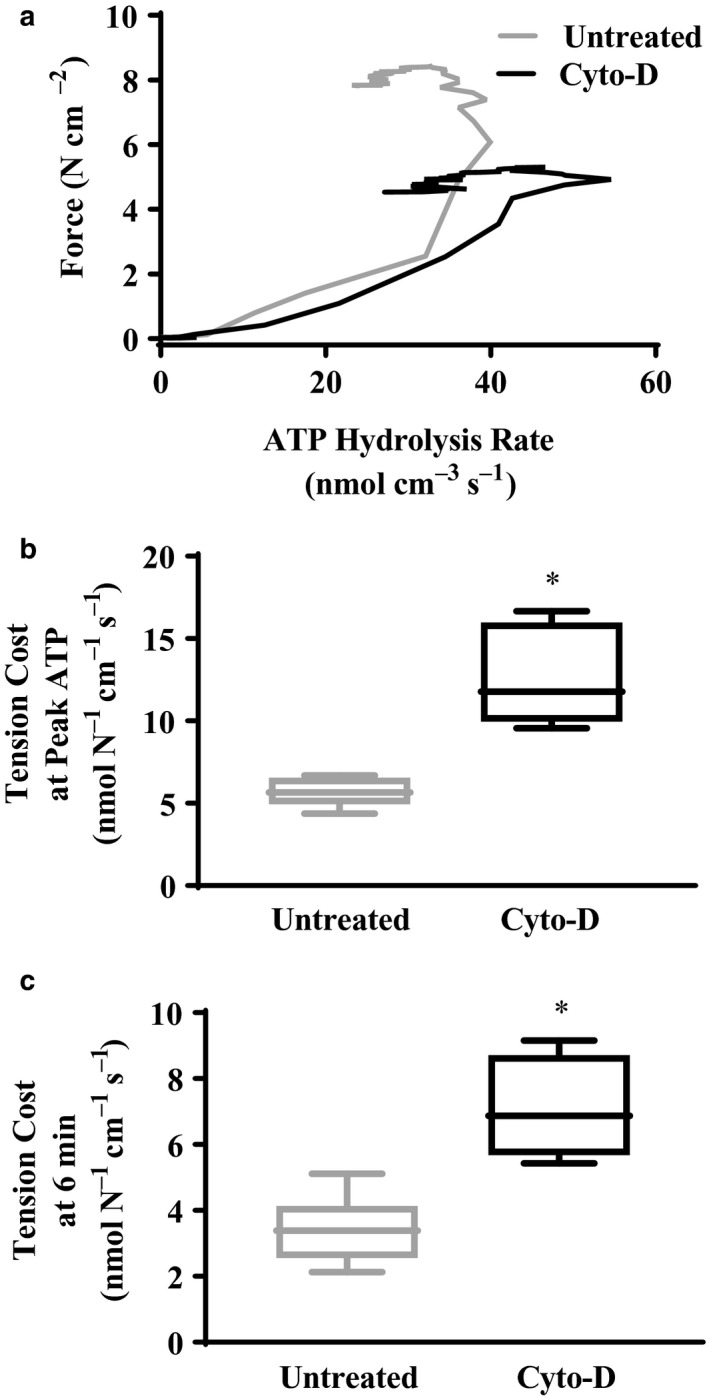
Effect of Cyto‐D on the relationship between isometric force generation and ATP hydrolysis rate was explored using phase‐loop plots. A representative plot is shown in (a). Tension cost was calculated as the ratio of isometric force and ATP hydrolysis rate and obviously varied across time after pCa 4.0 activation. Tension cost at the peak (b) and at 6 min (c) ATP hydrolysis rate in permeabilized porcine ASM strips during maximal Ca^2+^ activation (pCa 4.0) were both significantly increased after treatment with Cyto‐D. *Significant difference (*p* < .05) compared to untreated ASM strip (*n* = 6)

**Figure 6:**
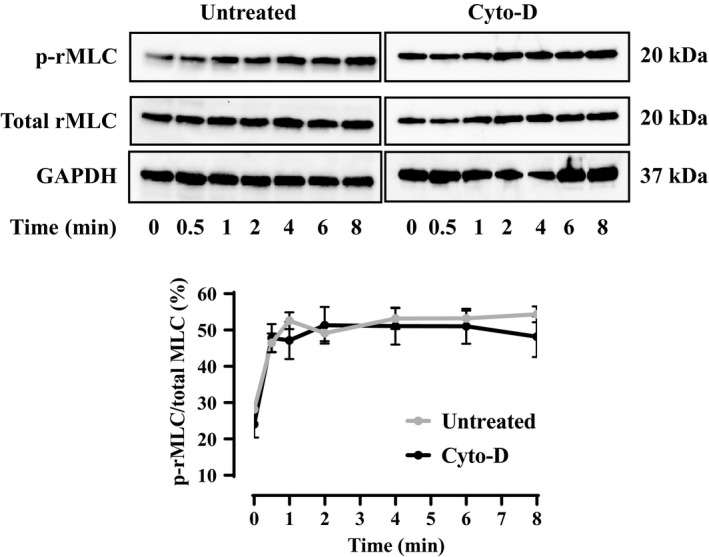
Effect of Cyto‐D on the extent of rMLC phosphorylation during maximal activation at a pCa 4.0. Representative western blots for p‐rMLC_20_ and total rMLC_20_ in permeabilized porcine ASM strips that were untreated or treated for 10 min to 1 µM Cyto‐D in pCa 9.0 solution followed by maximal Ca^2+^ activation (pCa 4.0) for 0, 0.5, 1, 2, 4, 6, and 8 min. In untreated ASM, rMLC_20_ phosphorylation increase after 0.5 min and stayed elevated after 1, 2, 4, 6, and 8 min. There was no significant difference on the extent of rMLC_20_ phosphorylation between Cyto‐D treated and untreated ASM strips (*n* = 6). GAPDH was used as loading control

During maximal activation at a pCa 4.0, isometric ATP hydrolysis rate in permeabilized porcine ASM strips also initially reached peak values at ∼1–2 min following activation then declined to lower levels throughout activation (Figure 4a). Interestingly, ATP hydrolysis (measured simultaneously with isometric force) preceded force development (Figures 3a and 4a). In permeabilized porcine ASM strips treated with Cyto‐D, the peak of ATP hydrolysis rate was significantly increased following pCa 4.0 activation compared to untreated ASM strips (*p* < .05, *n* = 6) (Figure 4b), whereas ATP hydrolysis rate values at 6 min were comparable in both Cyto‐D and untreated group (*p* > .05, *n* = 6) (Figure 4c). The dynamic relationship between isometric force and ATP hydrolysis rate was examined using a phase‐loop plot (Figure 5a). Compared to untreated permeabilized porcine ASM strips, the phase‐loop plots in ASM strips treated with Cyto‐D were shifted rightward. This indicated that for the same amount of force being generated, ATP hydrolysis rate was higher in ASM strips treated with Cyto‐D than in untreated ASM strips (Figure 4a). This result was supported by an increase in tension cost (ratio of ATP hydrolysis rate to force during pCa 4.0 activation) in permeabilized porcine ASM strips treated with Cyto‐D compared to untreated ASM strips at both the peak values (*p* > .05, *n* = 6) (Figure 5b) and 6 min after activation with pCa 4.0 (*p* > .05, *n* = 6) (Figure 5c).

The effect of Cyto‐D on rMLC phosphorylation during maximal activation (pCa 4.0 solution) was measured at various time points (0, 0.5, 2, 4, 6, and 8 min). The extent of rMLC phosphorylation was initially increased after 0.5 min in pCa 4.0 solution in both untreated and Cyto‐D treated ASM strips and remained elevated at 1, 2, 4, 6, and 4 min. The extent of rMLC phosphorylation was comparable in both untreated and Cyto‐D‐treated ASM strips for all time points (*n* = 6) (Figure 6).

## 4 DISCUSSION

In this study, we demonstrated that maximum isometric force in permeabilized porcine ASM (induced by pCa 4.0 activation) was reduced by inhibiting cytoskeletal remodeling, while ATP hydrolysis rate was increased. As a result, tension cost in ASM increased after Cyto‐D treatment due to a decrease in internal loading on cross‐bridge cycling and reduced external translation of force via actin‐myosin tethering the cortical cytoskeleton.

We and others have shown that in ASM actin and myosin content, cytoskeletal remodeling (actin/myosin polymerization) and the tethering of contractile elements through dense bodies to the cortical cytoskeleton and ASM membrane play an important role during sustained isometric force (Chitano et al., 2017; Dogan et al., 2017; Gunst & Fredberg, 2003; Gunst & Tang, 2000; Gunst et al., 2003; Gunst & Zhang, 2008; Jones, Perkins, et al., 1999; Mehta & Gunst, 1999; Sieck et al., 2019; Tang, 2018; Zhang & Gunst, 2008). In this study, we found that Cyto‐D, a potent inhibitor of actin polymerization, reduces porcine ASM force during maximal Ca^2+^ activation. These results are consistent with other studies using either phalloidin to stabilize F‐actin filaments in canine ASM during Ca^2+^‐induced activation or using Cyto‐D or latrunculin‐A to inhibit actin polymerization in canine tracheal smooth muscle activated with acetylcholine (ACh) (Jones, Perkins, et al., 1999; Mehta & Gunst, 1999). This reduction in ASM force following incubation with phalloidin or Cyto‐D can be attributed to either a decrease in the number of contractile elements or disruption of the tethering to the cortical cytoskeleton.

An important characteristic of smooth muscle is that during sustained isometric force, tension cost declines (Krisanda & Paul, 1988; Kuhn et al., 1990). This process has been attributed to the dephosphorylation of rMLC_20_ and the formation of slowly cycling latch bridges (Hai & Murphy, 1988a, 1988b). In the present study, we found that Cyto‐D increases tension cost in porcine ASM during maximal Ca^2+^ activation. These results are consistent with a previous study using phalloidin showing that tension cost, after an initial decline, increased throughout Ca^2+^ activation in canine ASM. However, phalloidin had no effect on rMLC_20_ phosphorylation (Jones, Lorenz, et al., 1999). In this study, we found that Cyto‐D had no effect on rMLC_20_ phosphorylation. Similarly, previous studies in canine tracheal smooth muscle have shown that latrunculin‐A had little effect on rMLC_20_ phosphorylation, while Cyto‐D only slightly decreased rMLC_20_ phosphorylation (Jones, Perkins, et al., 1999; Mehta & Gunst, 1999; Mehta, Tang, Wu, Atkinson, & Gunst, 2000). In addition, depletion of cytoskeletal proteins such as vinculin or focal adhesion protein paxillin involved in the tethering of actin filaments to the cortical cytoskeleton and ASM membrane reduces ASM force induced by ACh but does not affect rMLC_20_ phosphorylation (Opazo Saez et al., 2004; Tang et al., 2002). Taken together, these results suggest the dephosphorylation of rMLC_20_ cannot explain the observed decline in tension cost in ASM and that dynamic actin filament reorganization and the tethering of actin filaments to the cortical cytoskeleton is likely responsible for this process by affecting the internal load on cross‐bridges.

In conclusion, our results demonstrate that time‐dependent decrease in tension cost during is due to an increase in internal load on cross‐bridges by actin polymerization. Accordingly, actin polymerization not only transmits force to the extracellular matrix (i.e., cytoskeletal remodeling) from cycling cross‐bridges to cell membrane, but also increases the load on cross‐bridges thereby improving tension cost.

## 5 COMPETING INTERESTS

The authors have no conflicts of interests.

## AUTHOR CONTRIBUTIONS

PD, YSH, and GCS contributed to conception and design of the study. PD and YSH collected and analyzed data. PD, YSH, and GCS performed the statistical analysis. PD and GCS wrote the manuscript. All authors approved the final version of the manuscript and agree to be accountable for all aspects of the work in ensuring that questions related to the accuracy or integrity of any part of the work are appropriately investigated and resolved. All persons designated as authors qualify for authorship, and all those who qualify for authorship are listed.

## Data Availability

The datasets generated and analyzed in this study are available from the corresponding author on reasonable request. National Institutes of Health (NIH) HL126451 (GCS) and HL150890 (GCS).

## References

[phy214561-bib-0001] Chitano, P. , Wang, L. , Tin, G. Y. Y. , Ikebe, M. , Pare, P. D. , & Seow, C. Y. (2017). Smooth muscle function and myosin polymerization. Journal of Cell Science, 130, 2468–2480. 10.1242/jcs.202812 28596242

[phy214561-bib-0002] Dogan, M. , Han, Y. S. , Delmotte, P. , & Sieck, G. C. (2017). TNFalpha enhances force generation in airway smooth muscle. American Journal of Physiology Lung Cellular and Molecular Physiology, 312, L994–L1002. 10.1152/ajplung.00550.2016 28385814 PMC5495949

[phy214561-bib-0003] Fabiato, A. , & Fabiato, F. (1979). Calculator programs for computing the composition of the solutions containing multiple metals and ligands used for experiments in skinned muscle cells. Journal de Physiologie (Paris), 75, 463–505. 533865

[phy214561-bib-0004] Fredberg, J. J. , Jones, K. A. , Nathan, M. , Raboudi, S. , Prakash, Y. S. , Shore, S. A. , … Sieck, G. C. (1996). Friction in airway smooth muscle: Mechanism, latch, and implications in asthma. Journal of Applied Physiology (1985), 81, 2703–2703. 10.1152/jappl.1996.81.6.2703 9018525

[phy214561-bib-0005] Godt, R. E. , & Lindley, B. D. (1982). Influence of temperature upon contractile activation and isometric force production in mechanically skinned muscle fibers of the frog. Journal of General Physiology, 80, 279–297. 10.1085/jgp.80.2.279 6981684 PMC2228673

[phy214561-bib-0006] Gunst, S. J. , & Fredberg, J. J. (2003). The first three minutes: Smooth muscle contraction, cytoskeletal events, and soft glasses. Journal of Applied Physiology (1985), 95, 413–425. 10.1152/japplphysiol.00277.2003 12794100

[phy214561-bib-0007] Gunst, S. J. , & Tang, D. D. (2000). The contractile apparatus and mechanical properties of airway smooth muscle. European Respiratory Journal, 15, 600–616. 10.1034/j.1399-3003.2000.15.29.x 10759460

[phy214561-bib-0008] Gunst, S. J. , Tang, D. D. , & Opazo, S. A. (2003). Cytoskeletal remodeling of the airway smooth muscle cell: A mechanism for adaptation to mechanical forces in the lung. Respiratory Physiology & Neurobiology, 137, 151–168. 10.1016/s1569-9048(03)00144-7 14516723

[phy214561-bib-0009] Gunst, S. J. , & Zhang, W. (2008). Actin cytoskeletal dynamics in smooth muscle: A new paradigm for the regulation of smooth muscle contraction. American Journal of Physiology. Cell Physiology, 295, C576–C587. 10.1152/ajpcell.00253.2008 18596210 PMC2544441

[phy214561-bib-0010] Hai, C. M. , & Murphy, R. A. (1988). Cross‐bridge phosphorylation and regulation of latch state in smooth muscle. American Journal of Physiology, 254, C99–106. 10.1152/ajpcell.1988.254.1.C99 3337223

[phy214561-bib-0011] Hai, C. M. , & Murphy, R. A. (1988). Regulation of shortening velocity by cross‐bridge phosphorylation in smooth muscle. American Journal of Physiology, 255, C86–C94. 10.1152/ajpcell.1988.255.1.C86 3389402

[phy214561-bib-0012] Hirshman, C. A. , & Emala, C. W. (1999). Actin reorganization in airway smooth muscle cells involves Gq and Gi‐2 activation of Rho. American Journal of Physiology, 277, L653–L661. 10.1152/ajplung.1999.277.3.L653 10484474

[phy214561-bib-0013] Jones, K. A. , Lorenz, R. R. , Prakash, Y. S. , Sieck, G. C. , & Warner, D. O. (1999). ATP hydrolysis during contraction of permeabilized airway smooth muscle. American Journal of Physiology, 277, L334–342. 10.1152/ajplung.1999.277.2.L334 10444528

[phy214561-bib-0014] Jones, K. A. , Perkins, W. J. , Lorenz, R. R. , Prakash, Y. S. , Sieck, G. C. , & Warner, D. O. (1999). F‐actin stabilization increases tension cost during contraction of permeabilized airway smooth muscle in dogs. Journal of Physiology, 519(Pt 2), 527–538. 10.1111/j.1469-7793.1999.0527m.x 10457068 PMC2269509

[phy214561-bib-0015] Krisanda, J. M. , & Paul, R. J. (1988). Dependence of force, velocity, and O_2_ consumption on [Ca^2+^]o in porcine carotid artery. American Journal of Physiology, 255, C393–400. 10.1152/ajpcell.1988.255.3.C393 3421320

[phy214561-bib-0016] Kuhn, H. , Tewes, A. , Gagelmann, M. , Guth, K. , Arner, A. , & Ruegg, J. C. (1990). Temporal relationship between force, ATPase activity, and myosin phosphorylation during a contraction/relaxation cycle in a skinned smooth muscle. Pflugers Archiv. European Journal of Physiology, 416, 512–518. 10.1007/bf00382683 2146588

[phy214561-bib-0017] Mehta, D. , & Gunst, S. J. (1999). Actin polymerization stimulated by contractile activation regulates force development in canine tracheal smooth muscle. Journal of Physiology, 519(Pt 3), 829–840. 10.1111/j.1469-7793.1999.0829n.x 10457094 PMC2269534

[phy214561-bib-0018] Mehta, D. , Tang, D. D. , Wu, M. F. , Atkinson, S. , & Gunst, S. J. (2000). Role of Rho in Ca(2+)‐insensitive contraction and paxillin tyrosine phosphorylation in smooth muscle. American Journal of Physiology Cell Physiology, 279, C308–C318. 10.1152/ajpcell.2000.279.2.C308 10912996

[phy214561-bib-0019] Murphy, R. A. , & Rembold, C. M. (2005). The latch‐bridge hypothesis of smooth muscle contraction. Canadian Journal of Physiology and Pharmacology, 83, 857–864. 10.1139/y05-090 16333357 PMC2278007

[phy214561-bib-0020] Opazo Saez, A. , Zhang, W. , Wu, Y. , Turner, C. E. , Tang, D. D. , & Gunst, S. J. (2004). Tension development during contractile stimulation of smooth muscle requires recruitment of paxillin and vinculin to the membrane. American Journal of Physiology. Cell Physiology, 286, C433–447. 10.1152/ajpcell.00030.2003 14576084

[phy214561-bib-0021] Seow, C. Y. (2005). Myosin filament assembly in an ever‐changing myofilament lattice of smooth muscle. American Journal of Physiology Cell Physiology, 289, C1363–C1368. 10.1152/ajpcell.00329.2005 16275736

[phy214561-bib-0022] Seow, C. Y. , & An, S. S. (2020). The Force Awakens in the Cytoskeleton: The Saga of a Shape‐Shifter. American Journal of Respiratory Cell and Molecular Biology, 62, 550–551. 10.1165/rcmb.2019-0462ED 31940442 PMC7193797

[phy214561-bib-0023] Sieck, G. C. , Dogan, M. , Young‐Soo, H. , Osorio Valencia, S. , & Delmotte, P. (2019). Mechanisms underlying TNFalpha‐induced enhancement of force generation in airway smooth muscle. Physiol Rep, 7, e14220 10.14814/phy2.14220 31512410 PMC6739507

[phy214561-bib-0024] Sieck, G. C. , & Gransee, H. M. Respiratory muscles structure, function & regulation. San Rafael, Calif.: Morgan & Claypool Life Sciences, 2012, p. 1 online resource (viii, 87 p.). doi: 10.4199/C00057ED1V01Y2012ISP034.

[phy214561-bib-0025] Sieck, G. C. , Han, Y. S. , Pabelick, C. M. , & Prakash, Y. S. (2001). Temporal aspects of excitation‐contraction coupling in airway smooth muscle. Journal of Applied Physiology (1985), 91, 2266–2274. 10.1152/jappl.2001.91.5.2266 11641370

[phy214561-bib-0026] Sieck, G. C. , Han, Y. S. , Prakash, Y. S. , & Jones, K. A. (1998). Cross‐bridge cycling kinetics, actomyosin ATPase activity and myosin heavy chain isoforms in skeletal and smooth respiratory muscles. Comparative Biochemistry and Physiology Part B: Biochemistry and Molecular Biology, 119, 435–450. 10.1016/s0305-0491(98)00005-4 9734328

[phy214561-bib-0027] Sieck, G. C. , Kannan, M. S. , & Prakash, Y. S. (1997). Heterogeneity in dynamic regulation of intracellular calcium in airway smooth muscle cells. Canadian Journal of Physiology and Pharmacology, 75, 878–888. 10.1139/y97-103 9315357

[phy214561-bib-0028] Tang, D. D. (2018). The dynamic actin cytoskeleton in smooth muscle. Advances in Pharmacology, 81, 1–38. 10.1016/bs.apha.2017.06.001 29310796

[phy214561-bib-0029] Tang, D. D. , & Gunst, S. J. (2004). The small GTPase Cdc42 regulates actin polymerization and tension development during contractile stimulation of smooth muscle. Journal of Biological Chemistry, 279, 51722–51728. 10.1074/jbc.M408351200 15456777

[phy214561-bib-0030] Tang, D. D. , Wu, M. F. , Opazo Saez, A. M. , & Gunst, S. J. (2002). The focal adhesion protein paxillin regulates contraction in canine tracheal smooth muscle. Journal of Physiology, 542, 501–513. 10.1113/jphysiol.2002.021006 12122148 PMC2316150

[phy214561-bib-0031] Wang, Y. , Wang, R. , & Tang, D. D. (2020). Ste20‐like kinase‐mediated control of actin polymerization is a new mechanism for thin filament‐associated regulation of airway smooth muscle contraction. American Journal of Respiratory Cell and Molecular Biology, 62, 645–656. 10.1165/rcmb.2019-0310OC 31913659 PMC7193783

[phy214561-bib-0032] Zhang, W. , & Gunst, S. J. (2008). Interactions of airway smooth muscle cells with their tissue matrix: Implications for contraction. Proceedings of the American Thoracic Society, 5, 32–39. 10.1513/pats.200704-048VS 18094082 PMC2645300

